# Supplementary material for: MetaRibo-Seq measures translation in microbiomes
Source: Nat Commun. 2020 Jun 29;11:3268. doi: 10.1038/s41467-020-17081-z (PMC7324362; doi:10.1038/s41467-020-17081-z)
Supplement: Supplementary file 10 — Supplementary Data 7 [file 41467_2020_17081_MOESM10_ESM.zip › File2/Confidence_High_Taxonomy/221536_out.krona.html]

Javascript must be enabled to view this page.

members
magnitude
magnitudeUnassigned
count
unassigned
taxon
rank

221536\_out

30

2
1
superkingdom

SRS017191\_contig\_number\_contig-100\_1135.111761
30

1
phylum
1239

1
class
909932

1843488
order
1

family
1
909930

genus
1
33024


SRS144362\_contig\_number\_35568
33025
1
species

phylum
28
1224

28
class
1236

order
28
91347

family
543
6
28

SRS019129\_contig\_number\_14226SRS045244\_contig\_number\_contig-100\_3375.3376SRS077454\_contig\_number\_4959SRS077502\_contig\_number\_contig-100\_4673.4673SRS142890\_contig\_number\_15048SRS146996\_contig\_number\_contig-100\_7760.74238

genus
1
561
20

SRS140492\_contig\_number\_31229

19
species

SRS012273\_contig\_number\_44670SRS014683\_contig\_number\_3448SRS017521\_contig\_number\_47720SRS019068\_contig\_number\_contig-100\_5445.475054SRS046502\_contig\_number\_10769SRS049402\_contig\_number\_16187SRS057478\_contig\_number\_contig-100\_10922.10923SRS064276\_contig\_number\_contig-100\_9508.139063SRS075947\_contig\_number\_18376SRS076876\_contig\_number\_contig-100\_5089.5090SRS077646\_contig\_number\_contig-100\_1742.1743SRS098881\_contig\_number\_5475SRS142781\_contig\_number\_5848SRS143423\_contig\_number\_10182SRS144219\_contig\_number\_contig-100\_201.202SRS146888\_contig\_number\_15482SRS147022\_contig\_number\_13911SRS147151\_contig\_number\_12792SRS148196\_contig\_number\_7827
562

620
2
genus

624

SRS143014\_contig\_number\_contig-100\_382.383
1
species


SRS019601\_contig\_number\_3899
625
species
1
